# Efficacy and safety of alirocumab, a fully human PCSK9 monoclonal antibody, in high cardiovascular risk patients with poorly controlled hypercholesterolemia on maximally tolerated doses of statins: rationale and design of the ODYSSEY COMBO I and II trials

**DOI:** 10.1186/1471-2261-14-121

**Published:** 2014-09-20

**Authors:** Helen M Colhoun, Jennifer G Robinson, Michel Farnier, Bertrand Cariou, Dirk Blom, Dean J Kereiakes, Christelle Lorenzato, Robert Pordy, Umesh Chaudhari

**Affiliations:** University of Dundee, Dundee, Scotland, DD2 4BF UK; University of Iowa, Iowa City, IA USA; Point Médical, Dijon, France; L’Institut du Thorax, CHU de Nantes, Nantes, France; Division of Lipidology, Department of Medicine, University of Cape Town and MRC Cape Heart Group, Cape Town, South Africa; The Carl and Edyth Lindner Center for Research and Education at The Christ Hospital, Cincinnati, OH USA; Sanofi, Paris, France; Regeneron Pharmaceuticals, Inc., Tarrytown, NY USA; Sanofi, Bridgewater, NJ USA

**Keywords:** Alirocumab, Ezetimibe, Low-density lipoprotein cholesterol, Monoclonal antibody, Proprotein convertase subtilisin kexin type 9

## Abstract

**Background:**

Alirocumab is a fully human monoclonal antibody to proprotein convertase subtilisin kexin type 9 (PCSK9) under investigation for treatment of hypercholesterolemia and reduction of cardiovascular events.

**Methods/design:**

The COMBO studies, part of the Phase 3 ODYSSEY clinical trial program, are designed to evaluate the efficacy and safety of alirocumab as add-on therapy to stable, maximally tolerated daily statin, with or without other lipid-lowering therapy (LLT), in a planned 966 patients with hypercholesterolemia at high cardiovascular risk. COMBO I ( http://clinicaltrials.gov/show/NCT01644175) is placebo-controlled, with a double-blind treatment period of 52 weeks, and 306 planned patients who may receive other LLTs in addition to statin therapy. COMBO II ( http://clinicaltrials.gov/show/NCT01644188) has a double-blind treatment period of 104 weeks, comparing alirocumab with ezetimibe in 660 planned patients receiving statin therapy (but no other LLTs). The primary efficacy endpoint is the difference between treatment arms in percent change in low-density lipoprotein cholesterol (LDL-C) from baseline to week 24. Both studies utilized a starting dose of alirocumab 75 mg every 2 weeks (Q2W; administered as 1 mL solution via auto-injector). Patients with LDL-C levels ≥70 mg/dL after 8 weeks of treatment were up-titrated in a blinded manner at week 12 to alirocumab 150 mg Q2W (also 1 mL auto-injector).

**Discussion:**

In conclusion, the COMBO studies will provide information on the long-term efficacy and safety of alirocumab in high-risk patients when administered in addition to maximally tolerated statin therapy, with a flexible dosing strategy which allows for individualized therapy based on the degree of LDL-C lowering needed to achieve the desired treatment response.

**Trial registrations:**

COMBO I: NCT01644175 ( NCT01644175). COMBO II: NCT01644188 ( NCT01644188).

**Electronic supplementary material:**

The online version of this article (doi:10.1186/1471-2261-14-121) contains supplementary material, which is available to authorized users.

## Background

The benefits of lowering low-density lipoprotein cholesterol (LDL-C) levels on cardiovascular risk reduction for patients with and without coronary heart disease (CHD) are well established. For example, the 2012 Cholesterol Treatment Trialists’ meta-analysis (n = 174,149), including 22 controlled trials of standard statin regimens and five trials comparing more intensive versus less intensive statin regimens, showed a 21% reduction in cardiovascular disease (CVD) mortality and morbidity for every ~40 mg/dL (1 mmol/L) reduction in LDL-C, largely irrespective of age, sex, baseline LDL-C, or previous vascular disease [1]. The Cholesterol Treatment Trialists’ Collaboration also showed that there is further benefit from more intensive LDL-C lowering with statin therapy, even if LDL-C is already lower than ~80 mg/dL (2 mmol/L) at baseline [2].

European guidelines recommend LDL-C targets for patients based on their cardiovascular risk [3, 4]. In patients at very high risk, these guidelines recommend a LDL-C goal of ~70 mg/dL (<1.8 mmol/L) [3, 4], advocating a ≥50% reduction when target levels cannot be reached [3]. For intermediate- and high-risk patients, Canadian dyslipidemia guidelines recommend either a LDL-C level of ~77 mg/dL (≤2.0 mmol/L) or at least a 50% reduction in LDL-C [5]. The recently published 2013 American College of Cardiology (ACC)/American Heart Association (AHA) cholesterol management guidelines focus on intensity of statin treatment and recommend either high- or moderate-intensity statin therapy depending on cardiovascular risk [6]. High-intensity statin therapy (atorvastatin 40–80 mg/day or rosuvastatin 20–40 mg/day) generally reduces LDL-C levels by ≥50% while moderate-intensity statin therapy reduces LDL-by ≥30% to <50%. However, high-intensity statin therapy may be insufficient to achieve LDL-C targets when utilizing a ‘treat-to–target’ approach, may not be tolerated, or may be associated with a higher risk of myopathy [7]. Furthermore, despite the availability of other lipid-lowering therapies (LLTs) including cholesterol absorption inhibitors (ezetimibe), many high-risk patients with hypercholesterolemia still do not achieve adequate control of LDL-C levels and experience cardiovascular events [8–13]. Thus, treatment with other LDL-C-lowering therapies (either in combination with or without statin therapy) may be warranted to achieve better LDL-C control. Indeed, the 2013 ACC/AHA guideline recommends consideration of non-statin drug therapy for high-risk individuals needing additional LDL-C lowering, such as those with clinical atherosclerotic CVD, untreated LDL-C ≥190 mg/dL, or diabetes aged 40–75 years [6]. However, it should be noted that, to date, major trials of these hypolipidemic drugs in combination with statins have failed to show any additional improvement in cardiovascular outcomes as compared with statins alone [14, 15].

Proprotein convertase subtilisin kexin type 9 (PCSK9) is the ninth member of the proprotein convertase family [16]. PCSK9 inhibitors protect hepatic LDLRs against PCSK9-mediated degradation and, consequently, reduce plasma levels of LDL-C [17]. PCSK9 inhibition has the potential to provide a complementary mechanism to significantly reduce LDL-C beyond what is possible with statins, since statins are known to increase PCSK9 levels at transcriptional level [18]. Several approaches to PCSK9 inhibition are in development, the most advanced being monoclonal antibodies (mAbs), which prevent PCSK9 from binding to the LDLR (reviewed in Farnier, [19] and Cariou et al. [20]).

Alirocumab is a fully human mAb to PCSK9 that has shown considerable promise in Phase 2 trials [21–23] and is currently in Phase 3 trials for the treatment of hypercholesterolemia and reduction of cardiovascular events.

The COMBO I (http://clinicaltrials.gov/show/NCT01644175) and II (http://clinicaltrials.gov/show/NCT01644188) trials form part of the alirocumab Phase 3 ODYSSEY clinical trial program, which currently comprises 14 studies across a range of patient groups and clinical settings involving more than 23,500 planned patients, to further assess the efficacy and safety of alirocumab. The COMBO studies have been specifically designed to evaluate the long-term efficacy and safety of alirocumab as add-on therapy to stable, maximally tolerated, daily statin therapy in patients with hypercholesterolemia at high cardiovascular risk. COMBO I compares alirocumab against placebo, with patients permitted to receive other stable doses of LLTs in addition to maximally tolerated daily statin therapy. COMBO II compares alirocumab versus ezetimibe when administered in conjunction with statin therapy only (i.e. other LLTs are not allowed in COMBO II).

## Methods

### Study design

**Figure 1:**
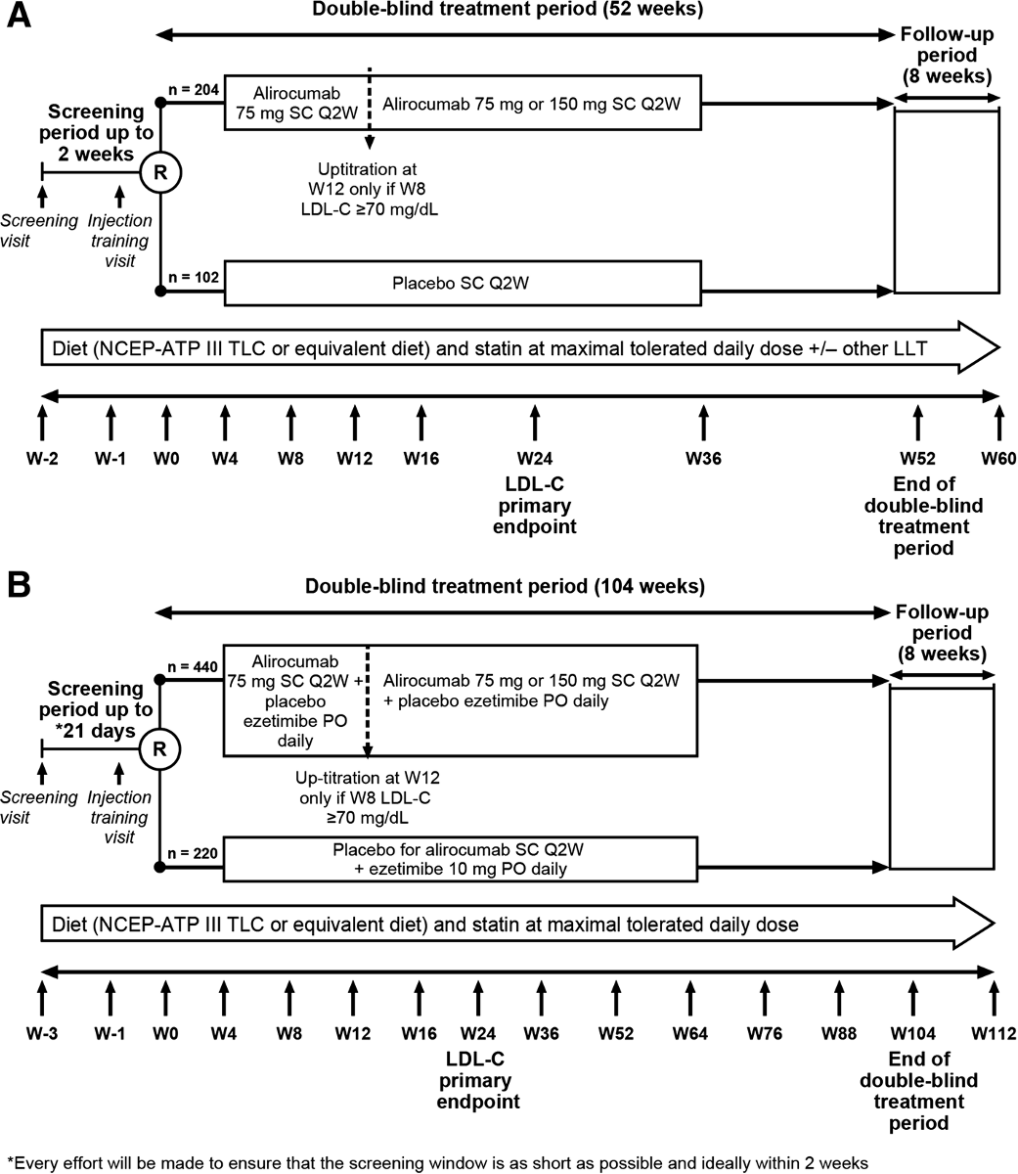
**Study design. A)** ODYSSEY COMBO I. **B)** ODYSSEY COMBO II. LDL-C, low-density lipoprotein cholesterol; LLT, lipid-lowering therapy; NCEP ATP III, National Cholesterol Education Program Adult Treatment Panel III; PO, *per os*; Q2W, every 2 weeks; R, randomization; SC, subcutaneous; TLC, therapeutic lifestyle changes.

COMBO I is a Phase 3, randomized, double-blind, placebo-controlled, parallel-group, multicenter, 52-week study being conducted at 76 sites in the USA. This study evaluates the efficacy and safety of alirocumab as add-on therapy to stable, maximally tolerated doses of daily statin, with or without other stable LLT, in a planned population of 306 high-risk patients with poorly controlled hypercholesterolemia (Figure 1A). COMBO I began screening patients in July 2012 and completed collection of data in April 2014 (http://clinicaltrials.gov/show/NCT01644175); data analyses are ongoing.COMBO II is a Phase 3, randomized, double-blind, active-controlled, parallel-group, multinational 104-week study being conducted at 126 sites in Europe, Israel, North America, South Africa, and South Korea. The planned population size is 660 high-risk patients with poorly controlled hypercholesterolemia on stable, maximally tolerated daily statin therapy. This study began screening patients in August 2012 and is anticipated to complete in July 2015 (http://clinicaltrials.gov/show/NCT01644188). Unlike COMBO I, with a placebo arm, COMBO II incorporates an active-treatment arm (ezetimibe) with double-dummy design (Figure 1b) and patients are not allowed to receive any other LLTs besides statin and their randomized treatment.

The studies are being conducted in compliance with the principles laid down by the 18th World Medical Assembly (Helsinki, 1964) and all applicable amendments laid down by the World Medical Assemblies and according to the International Conference on Harmonization Guidelines for Good Clinical Practice. The protocols have been reviewed and approved by the institutional review board of each participating center (Additional file 1). All participants have provided written informed consent.

### Inclusion and exclusion criteria

Principal inclusion and exclusion criteria are shown in Table 1; full inclusion and exclusion criteria for both studies can be found in Additional file 1.

**Table 1:** Key inclusion and exclusion criteria*

Inclusion criteria ^†^	
COMBO I	COMBO II
Patients with hypercholesterolemia and established CHD or CHD risk equivalents^#^ with LDL-C poorly controlled with a maximally tolerated daily dose of statin **with or without other LLT, both at stable dose for at least 4 weeks (6 weeks for fenofibrate) prior to the screening visit (week −2)**	Patients with hypercholesterolemia and established CHD or CHD risk equivalents^#^ with LDL-C poorly controlled with a maximally tolerated daily dose of statin **at stable dose for at least 4 weeks prior to the screening visit (week −3)**
Baseline entry criteria: LDL-C levels depending on history of documented CVD:	Baseline entry criteria: LDL-C levels depending on history of documented CVD:
• LDL-C ≥70 mg/dL (≥1.81 mmol/L) at the screening visit with a history of documented CVD	• LDL-C ≥70 mg/dL (≥1.81 mmol/L) at the screening visit with a history of documented CVD
• LDL-C ≥100 mg/dL (≥2.59 mmol/L) at the screening visit in patients without history of documented CVD	• LDL-C ≥100 mg/dL (≥2.59 mmol/L) at the screening visit in patients without history of documented CVD
**Exclusion criteria** ^**†**^	
**COMBO I**	**COMBO II**
Age <18 years	Age <18 years
Fasting serum triglycerides >400 mg/dL (>4.52 mmol/L) during the screening period	Fasting serum triglycerides >400 mg/dL (>4.52 mmol/L) during the screening period
Currently taking a statin that is not simvastatin, atorvastatin, or rosuvastatin taken daily at a registered dose	Currently taking a statin that is not simvastatin, atorvastatin, or rosuvastatin taken daily at a registered dose
**Use of fibrates, other than fenofibrate, within 6 weeks prior to the screening visit (week −2)**	**Use of concomitant meds**
	• **Ezetimibe, omega-3 fatty acid (at doses ≥1,000 mg daily), nicotinic acid, bile acid-binding sequestrant, or red yeast rice products in the past 4 weeks prior to screening visit (week −3)**
	• **Use of fibrates in the past 6 weeks prior to screening visit (week −3)**

CHD, coronary heart disease; CVD, cardiovascular disease (defined as CHD, ischemic stroke or peripheral arterial disease – see Additional file 1 for additional details); LDL-C, low-density lipoprotein cholesterol; LLT, lipid-lowering therapy.

*Further information on the inclusion and exclusion criteria can be found in Additional file 1.

^†^Differences between COMBO I and COMBO II are shown in bold font.

^#^See Additional file 1 for definition of CHD risk equivalents.

All patients in COMBO I and II have hypercholesterolemia and established CHD or CHD risk equivalents, with LDL-C poorly controlled with a maximally tolerated daily dose of statin. In COMBO I only, patients were also permitted to receive other LLT on top of statin, provided both the statin and other LLT were at stable dose for at least 4 weeks (6 weeks for fenofibrate) prior to the screening visit; in COMBO II, statin dose was required to be stable for at least 4 weeks prior to the screening visit and other LLTs were not permitted. At screening, patients either had LDL-C ≥70 mg/dL (≥1.81 mmol/L) with documented CVD or LDL-C ≥100 mg/dL (≥2.59 mmol/L) with no documented history of CVD (Table 1).

The ‘maximum tolerated dose’ of statin was defined as either rosuvastatin 20 mg or 40 mg daily, atorvastatin 40 mg or 80 mg daily, or simvastatin 80 mg daily (if already on this dose for >1 year). However, patients not able to tolerate the above statin doses remained eligible for inclusion if they were on a lower dose of daily atorvastatin, rosuvastatin, or simvastatin provided that the investigator had a documented reason for not using the higher dose (Additional file 1).

### Study procedures

Patients meeting the inclusion criteria entered a screening period of up to 2 (COMBO I) or 3 (COMBO II) weeks prior to randomization. During screening, patients completed informed consent, inclusion/exclusion criteria were further assessed, patient information was collected, and patients were trained in the use of the auto-injector device. In addition, vital signs were taken, a 12-lead electrocardiogram was performed, and fasting blood and urine samples were obtained for analysis. AEs will be assessed from the screening visit throughout the study.

LDL-C will be calculated using the Friedewald formula at screening and at all time points during the double-blind treatment periods. If TGs exceed 400 mg/dL (4.52 mmol/L) then the central laboratory will reflexively measure LDL-C (via the beta quantification method) rather than calculating it. LDL-C will also be measured (via the beta quantification method) at week 0 and week 24. Other lipid parameters, including total cholesterol, HDL-C, TGs, Apo B, Apo A1, and Lp(a), will be measured directly by the central laboratory.

### COMBO I

Eligible patients were randomized (2:1 alirocumab:placebo), with stratification by 1) prior history of myocardial infarction (MI) or ischemic stroke, and 2) intensity of statin treatment, to ensure balance between arms for these factors. After randomization, patients entered a double-blind treatment period of 52 weeks. In addition to existing statin and other existing LLT if appropriate, patients randomized to alirocumab received a 75 mg subcutaneous (SC) dose every 2 weeks (Q2W), administered as a single 1 mL injection utilizing an auto-injector, from randomization to week 12. Patients randomized to placebo received a 1 mL SC placebo injection from an identical auto-injector.

At week 12, patients randomized to alirocumab were up-titrated to 150 mg Q2W if the week 8 LDL-C was ≥70 mg/dL (1.81 mmol/L). To maintain blinding, the patient and investigator were not informed of the week 8 LDL-C levels (or any lipid values after randomization); continuation or up-titration of dose occurred in an automated and blinded manner. The 150 mg Q2W dose of alirocumab was also administered as a 1 mL solution in an auto-injector.

On-site patient assessments during the treatment period were scheduled at randomization and then weeks 4, 8, 12, 16, 24, 36, and 52 (end of treatment visit) (Figure 1). After the treatment period, there will be an 8-week follow-up period.

### COMBO II

Eligible patients were randomized (2:1 alirocumab:ezetimibe), with stratification for 1) prior history of MI or ischemic stroke, 2) intensity of statin treatment, and 3) geographic region, to ensure balance between arms in these factors. After randomization, patients entered a double-blind, double-dummy treatment period of 104 weeks. Patients were randomized to either alirocumab 75 mg SC Q2W plus placebo for ezetimibe *per os* (PO) daily or placebo for alirocumab SC Q2W plus ezetimibe 10 mg PO daily. At week 12, patients randomized to alirocumab were up-titrated to 150 mg Q2W if the week 8 LDL-C was ≥70 mg/dL (1.81 mmol/L).

On-site patient assessments were scheduled at regular intervals from randomization to week 104 (end of treatment visit) (Figure 1). After the treatment period, there will be an 8-week follow-up period.

In both studies, patients were asked to remain on a stable diet (National Cholesterol Education Program Adult Treatment Panel III therapeutic lifestyle changes diet or equivalent) and the daily statin dose should be stable throughout the entire study duration from screening to the follow-up visit. Modification to the statin (and, in the case of COMBO I, other background LLT) is only allowed under special circumstances.

### Endpoints and assessments

The primary objective of both studies is to demonstrate reduction of calculated LDL-C by alirocumab as add-on therapy to stable maximally tolerated daily statin, either (a) with or without other LLTs, in comparison with placebo (COMBO I) or (b) in comparison with ezetimibe 10 mg daily (COMBO II). The primary endpoint for both studies is the difference between arms in percent change in calculated LDL-C from baseline to week 24, using all LDL-C values regardless of adherence to treatment (intent-to-treat [ITT] approach). The key secondary efficacy endpoints are very similar in the two studies and are summarized in Table 2.

**Table 2:** **Primary and key secondary endpoints in COMBO I and II**

Primary endpoint	Population
Percentage change in calculated LDL-C from baseline to week 24 in the ITT population, using all LDL-C values regardless of adherence to treatment (ITT analysis)	ITT
**Key secondary endpoints**	**Population**
Percentage change in calculated LDL-C from baseline to week 24 in the modified ITT population, using all LDL-C values during the efficacy treatment period (on-treatment analysis)	mITT
Percentage change in calculated LDL-C from baseline to week 12 (ITT analysis)	ITT
Percentage change in calculated LDL-C from baseline to week 12 (on-treatment analysis)	mITT
Percentage change in Apo B from baseline to week 24 (ITT analysis)	ITT
Percentage change in Apo B from baseline to week 24 (on-treatment analysis)	mITT
Percentage change in non-HDL-C from baseline to week 24 (ITT analysis)	ITT
Percentage change in non-HDL-C from baseline to week 24 (on-treatment analysis)	mITT
Percentage change in total cholesterol from baseline to week 24 (ITT analysis)	ITT
Percentage change in Apo B from baseline to week 12 (ITT analysis)	ITT
Percentage change in non-HDL-C from baseline to week 12 (ITT analysis)	ITT
Percentage change in total cholesterol from baseline to week 12 (ITT analysis)	ITT
Percentage change in calculated LDL-C from baseline to week 52 (ITT analysis)	ITT
Proportion of patients reaching calculated LDL-C <70 mg/dL (1.81 mmol/L) at week 24 (ITT analysis)	ITT
Proportion of patients reaching calculated LDL-C <70 mg/dL (1.81 mmol/L) at week 24 (on-treatment analysis)	mITT
Percentage change in Lp(a) from baseline to week 24 (ITT analysis)	ITT
Percentage change in HDL-C from baseline to week 24 (ITT analysis)	ITT
Percentage change in fasting TGs from baseline to week 24 (ITT analysis)	ITT
Percentage change in Apo A1 from baseline to week 24 (ITT analysis)	ITT
Percentage change in Lp(a) from baseline to week 12 (ITT analysis)	ITT
Percentage change in HDL-C from baseline to week 12 (ITT analysis)	ITT
Percentage change in fasting TGs from baseline to week 12 (ITT analysis)	ITT
Percentage change in Apo A1 from baseline to week 12 (ITT analysis)	ITT

Apo, apolipoprotein; HDL-C, high-density lipoprotein cholesterol; ITT, intent-to-treat; LDL-C, low-density lipoprotein cholesterol; Lp(a), lipoprotein (a); mITT modified intent-to-treat; TGs, triglycerides.

Safety will be assessed throughout the duration of the treatment periods by AE reporting (including adjudicated cardiovascular events), laboratory analyses, and vital signs measurement. Since the long-term effects of PCSK9 inhibition on top of a statin in humans are unknown, a number of AEs are defined as being of special interest and will be monitored (Additional file 1).

### Statistical analyses

#### Sample size determination

In COMBO I, a sample size of 45 patients (30 in alirocumab, 15 in placebo) was determined to have 95% power to detect a difference in mean percentage change in LDL-C of 30% with a 0.05 two-sided significance level, assuming a common standard deviation of 25% and all 45 patients having an evaluable primary endpoint. Meanwhile, in COMBO II, a sample size of 96 patients (64 in alirocumab, 32 in ezetimibe) was determined to have 95% power to detect a difference in mean percentage change in LDL-C of 20% with a 0.05 two-sided significance level, assuming common standard deviation of 25% and all 96 patients having an evaluable primary endpoint.

However, to meet regulatory requirements across the overall ODYSSEY Program, sample sizes were increased in most of the alirocumab Phase 3 studies to assess the safety of alirocumab appropriately in the overall integrated safety database. Therefore, the final total sample sizes were increased to 306 patients in COMBO I and 660 in COMBO II, both with a randomization ratio of 2:1.

#### Primary analysis

The primary efficacy analysis population will be the ITT population, comprising all randomized patients with at least one baseline calculated LDL-C value available and at least one calculated LDL-C value available at one of the planned time points from weeks 4 to 24 (regardless of treatment adherence).

The percentage change in calculated LDL-C from baseline to week 24 will be analyzed using a mixed effect model with repeated measures (MMRM) approach to account for missing data [24, 25]. All available post-baseline data at planned time points from week 4 to 52 regardless of status on- or off-treatment will be used in the MMRM for the ITT analysis, with the model used to provide least-squares means estimates and comparison between treatment arms of LDL-C reductions at week 24. The models will include fixed categorical effects of treatment group, randomization strata, time point, treatment-by-time point interaction, and strata-by-time point interaction, as well as the continuous fixed covariates of baseline LDL-C value and baseline value-by-time point interaction. Although both trials have the LDL-C differences at week 24 as the primary endpoint, they extend beyond 24 weeks so as to maximize available safety data and to generate further data on durability of lipid lowering effects. The studies will extend to the planned duration regardless of any efficacy data from the week 24 timepoint.

#### Secondary analysis

A hierarchical procedure will be used to control type I error and handle multiple secondary endpoint analyses. If the primary endpoint analysis (ITT) is significant at 5% alpha level, key secondary efficacy endpoints will be tested sequentially in the order given in Table 2. In particular, LDL-C reduction at week 24 will be analyzed ‘on-treatment’ in the modified ITT (mITT) population if the primary analysis is significant in the ITT population.

The mITT population will exclude those patients from the ITT population who do not have a calculated LDL-C value available while on-treatment (defined as the period between first dose of study treatment and up to 21 days after last injection, or 3 days after last capsule intake, whichever came first). For the on-treatment analysis, all available on-treatment measurements (i.e. up to 21 days after last injection/3 days after last capsule, whichever comes first) at planned time points from weeks 4 to 52 will be used in the MMRM.

Continuous secondary endpoints, except Lp(a) and TGs, will be analyzed using the same MMRM model as for the primary endpoint. Lp(a) and TGs (which have a non-Gaussian distribution) and the binary secondary endpoints (proportion of patients with LDL-C <70 mg/dL and <100 mg/dL) will be analyzed using a multiple imputation approach for handling of missing values followed by robust regression (for Lp[a] and TGs) or logistic regression (for the binary endpoints).

#### Safety analysis

AEs (including adjudicated cardiovascular events), laboratory parameters, and vital signs will be reported descriptively, based on the safety population (all randomized patients who received at least one dose or partial dose of study treatment). The safety analysis will focus on the treatment-emergent AE period, defined as the time from the first double-blind dose to the last double-blind dose of the investigational product + 70 days (10 weeks). The studies are not powered to assess the impact on cardiovascular outcomes, which will be assessed in a separate, large outcomes study (http://clinicaltrials.gov/show/NCT01663402) [26].

## Discussion

Prior clinical studies and observational analyses have demonstrated that many patients may struggle to achieve effective control of their LDL-C levels utilizing existing LLTs, with high baseline LDL-C levels, efficacy limitations, intolerance, and poor compliance all contributing factors [27–32]. However, the addition of a mAb targeting PCSK9 to existing LLT may help those patients at high cardiovascular risk to achieve the recommended LDL-C levels or percentage lowering. The ODYSSEY COMBO studies are designed to assess the efficacy and safety of alirocumab as add-on therapy to stable, maximally tolerated daily statin therapy in patients with hypercholesterolemia at high cardiovascular risk versus placebo (COMBO I) or ezetimibe (COMBO II). These patients, at high cardiovascular risk, are recommended for intensive lowering of LDL-C.

COMBO II will also allow a comparison of the efficacy and safety of alirocumab versus ezetimibe, both given on top of maximally tolerated doses of statin. Ezetimibe is frequently added to statins to provide greater reductions in LDL-C, particularly where patients are unable to tolerate titration to a higher potency [33]. However, while generally well tolerated, ezetimibe lowers LDL-C levels only modestly. As an add-on to statin therapy, a 15.1% greater reduction in LDL-C was observed with statin + ezetimibe combination therapy when compared with statin monotherapy in a meta-analysis of 27 double-blind, placebo-controlled, or active comparative studies of over 21,000 subjects with a mean treatment duration of 9 weeks [34]. In this meta-analysis, only 10.3% of patients with established CHD who received statin monotherapy achieved the pre-defined LDL-C goal of <70 mg/dL. Even with the addition of ezetimibe, only 32.1% of patients achieved this LDL-C goal, suggesting the need for more effective LLT [34].

The trials within the ODYSSEY program use a treat-to-goal approach and are designed to address unmet needs of patient populations on current standard of care unable to achieve LDL-C goals, using a flexible dosing strategy for individualized therapy based on degree of LDL-lowering needed to achieve an adequate treatment response. A key aspect of the COMBO studies is the potential to up-titrate alirocumab-treated patients based on their LDL-C levels after 8 weeks of treatment. The starting dose of 75 mg Q2W was selected to provide an approximate 50% decrease in LDL-C from baseline when added to statin therapy, as determined by a dose-response model [35]. As such, all patients were initially treated with 75 mg Q2W. However, those patients whose LDL-C levels remain ≥70 mg/dL after 8 weeks of treatment were dose up-titrated in a blinded manner at week 12 to 150 mg Q2W without a need to increase the injection volume. With this flexible treatment scheme, most patients can be expected to achieve an LDL-C level of <70 mg/dL without reaching very low LDL-C levels. However, there is the potential that some patients may achieve LDL-C levels of below 25 mg/dL and reducing LDL-C to such very low levels has been controversial with respect to cancers and hemorrhagic stroke risks. Several observational studies have suggested an association between hemorrhagic stroke and low serum cholesterol [36–38]. Consistent with this, the Cholesterol Treatment Trialists' meta-analysis [2] found an excess of hemorrhagic stroke in the meta analysis of more versus less intensive statin regimes, though the excess risk was 50 times less than the beneficial effect on occlusive stroke. No association was found between intensive statin therapy and cancer risk [2]. Nonetheless, since data from patients achieving very low levels of LDL-C are sparse, additional monitoring by the Data Monitoring Committee will be implemented for those patients reaching LDL-C levels of <25 mg/dL to further evaluate the safety of very low LDL-C levels.

The COMBO trials have used differing lipid entry criteria depending on whether entrants had a clinical history of CVD or were CHD risk equivalent. This reflected that at the time of design, the revised ATPIII then operant in the US did not include an unequivocal recommendation of a target <70 mg/dL in all such patients, but left it as a “therapeutic option” reflecting some degree of uncertainty [39]. The new 2013 ACC/AHA guidelines have moved away from citing lipid targets but instead focus on the intensity of statin therapy being tailored to CVD risk [6]. Most of the entrants to the COMBO trials would be eligible for intensive therapy under these new guidelines. Regardless of whether physicians are working to these 2013 ACC/AHA guidelines [6] or guidelines that continue to use LDL-C targets [40], the data the COMBO trial will provide on the efficacy and safety of alirocumab in high-risk patients when administered in addition to maximally tolerated statin therapy will be useful since patients warranting intensive statin therapy may not tolerate it.

Overall, the ODYSSEY program comprises 14 studies of more than 23,500 planned subjects across more than 2,000 study centers worldwide. The program will evaluate multiple patient populations (including patients at high cardiovascular risk, patients with heterozygous familial hypercholesterolemia, and patients with well-defined statin intolerance) and different treatment options (including alirocumab monotherapy, combination therapy with statins and other LLTs, and flexible dosing options). These studies follow a robust approach to investigate a new class of drugs with a novel mechanism of action, with efficacy and safety studies ranging from 24–104 weeks duration (rather than 12–52 weeks) to provide a greater amount of double-blind safety data for building confidence in alirocumab as a potential therapeutic option. Of note, the ODYSSEY program also includes a large cardiovascular outcomes study (http://clinicaltrials.gov/show/NCT01663402) [26] which will determine the long-term impact of alirocumab and lower levels of LDL-C on the occurrence of cardiovascular events in 18,000 patients after a recent (<52 weeks) acute coronary syndrome event, with a randomized treatment period of 64 months.

In summary, the COMBO studies are the longest duration placebo/ezetimibe-controlled trials of a PCSK9 inhibitor in high-risk patients with poorly controlled LDL-C on maximum tolerated standard of care. They will help to guide clinical decision making on the next LLT to use beyond statin therapy.

## Electronic supplementary material

Additional file 1: **Additional study details.** (DOC 84 KB)

## Abbreviations

ACC: American College of Cardiology
AE: Adverse event
AHA: American Heart Association
Apo: Apolipoprotein
CHD: Coronary heart disease
CVD: Cardiovascular disease
HDL-C: High-density lipoprotein cholesterol
ITT: Intent-to-treat
LDL-C: Low-density lipoprotein cholesterol
LDLR: Low-density lipoprotein receptor
LLT: Lipid-lowering therapy
Lp(a): Lipoprotein (a)
mAB: Monoclonal antibody
MI: Myocardial infarction
mITT: Modified ITT
MMRM: Mixed effect model with repeated measures
PCSK9: Proprotein convertase subtilisin kexin type 9
PO: Per os
Q2W: Every 2 weeks
SC: Subcutaneous
TG: Triglyceride.
