# Supplementary material for: Efficacy and safety of alirocumab, a fully human PCSK9 monoclonal antibody, in high cardiovascular risk patients with poorly controlled hypercholesterolemia on maximally tolerated doses of statins: rationale and design of the ODYSSEY COMBO I and II trials
Source: BMC Cardiovasc Disord. 2014 Sep 20;14:121. doi: 10.1186/1471-2261-14-121 (PMC4190302; doi:10.1186/1471-2261-14-121)
Supplement: Supplementary file 1 — Additional file 1: Additional study details. (DOC 84 KB) [file 12872_2014_776_MOESM1_ESM.doc]

# Appendix. Additional Study Details

## COMBO I

### Full inclusion criteria

Patients with hypercholesterolemia and established coronary heart disease (CHD) or CHD risk equivalents (see below for definitions) who have poorly controlled low-density lipoprotein cholesterol (LDL-C) with a maximally tolerated daily dose of statin with or without other lipid-lowering therapy (LLT), both at stable dose for at least 4 weeks prior to the screening visit (and 6 weeks for fenofibrate prior to the screening visit). Signed written informed consent will be obtained from all patients.

Definition of maximally tolerated dose (any of the following are acceptable): rosuvastatin 20 mg or 40 mg daily, atorvastatin 40 mg or 80 mg daily, or simvastatin 80 mg daily (if already on this dose for >1 year ). Patients not able to be on any of the above statin doses should be treated with the dose of daily atorvastatin, rosuvastatin, or simvastatin which is considered appropriate for the patient as per the investigator's judgment or concerns. Some examples of acceptable reasons for a patient taking a lower statin dose include: adverse effects on higher doses, advanced age, low body mass index (BMI), regional practices, local prescribing information, concomitant medications, and comorbid conditions such as impaired glucose tolerance/impaired fasting glucose.

#### Established CHD

This is defined as a documented history of CHD, including one or more of the following: acute myocardial infarction (MI), silent MI, unstable angina, coronary revascularization procedure (e.g., percutaneous coronary intervention [PCI] or coronary artery bypass graft [CABG] surgery), and clinically significant CHD diagnosed by invasive or non-invasive testing (such as coronary angiography, stress test using treadmill, stress echocardiography, or nuclear imaging).

#### CHD risk equivalents

The definition of CHD risk equivalents includes one or more of the following four criteria:

1. Documented peripheral arterial disease (PAD) (one of the following criteria [a, b, or c] must be satisfied):
   1. Current intermittent claudication (muscle discomfort in the lower limb produced by exercise that is both reproducible and relieved by rest within 10 minutes) of presumed atherosclerotic origin *together with* ankle-brachial index equal to or less than 0.90 in either leg at rest OR
   2. History of intermittent *together with* endovascular procedure or surgical intervention in one or both legs because of atherosclerotic disease OR
   3. History of critical limb ischemia *together with* thrombolysis, endovascular procedure or surgical intervention in one or both legs because of atherosclerotic disease.
2. Documented ischemic stroke with a focal ischemic neurological deficit that persisted more than 24 hours, considered as being of atherothrombotic origin. Computed tomography (CT) or magnetic resonance imaging (MRI) must have been performed to rule out hemorrhage and non-ischemic neurological disease.
3. Documented chronic kidney disease (CKD) as defined by estimated glomerular filtration rate (eGFR) of ≥30‒<60 ml/min/1.73 m^2^ for 3 months or more, including the screening visit.
4. Known history of diabetes mellitus *and* two or more additional risk factors, including:
   1. History of hypertension (established on antihypertensive medication).
   2. Documented history of ankle-brachial index ≤0.90.
   3. Documented history of microalbuminuria or macroalbuminuria *or* dipstick urinalysis at screening visit (week -2) with >2+ protein.
   4. Documented history of preproliferative or proliferative retinopathy or laser treatment for retinopathy.
   5. Known family history of premature CHD (CHD in father or brother before 55 years of age; CHD in mother or sister before 65 years of age).

### Full exclusion criteria

Patients who have met all the above inclusion criteria will be screened for the following exclusion criteria.

#### Exclusion criteria related to study methodology

Patients without established CHD or CHD risk equivalents; LDL-C <70 mg/dl (<1.81 mmol/L) at the screening visit in patients with an history of documented cardiovascular disease [CVD]); LDL-C <100 mg/dL (<2.59 mmol/L) at the screening visit in patients without history of documented CVD); those not on a stable dose of LLT (including statin) for at least 4 weeks and/or fenofibrate for at least 6 weeks, as applicable, prior to the screening visit and from screening to randomization; currently taking a statin other than simvastatin, atorvastatin, or rosuvastatin; simvastatin, atorvastatin, or rosuvastatin is not taken daily or not taken at a registered dose; daily doses above atorvastatin 80 mg, rosuvastatin 40 mg, or simvastatin 40 mg (except for patients on simvastatin 80 mg for >1 year, who are eligible); use of fibrates, other than fenofibrate within 6 weeks of the screening visit or between screening and randomization visits; use of nutraceutical products or over-the-counter therapies that may affect lipids which have not been at a stable dose/amount for at least 4 weeks prior to the screening visit or between screening and randomization visits; use of red yeast rice products within 4 weeks of the screening visit or between screening and randomization visits; patients who have received plasmapheresis treatment within 2 months prior to the screening visit (week -2), or have plans to receive it during the study; recent (within 3 months prior to the screening visit or between screening and randomization visits) MI, unstable angina leading to hospitalization, PCI, CABG, uncontrolled cardiac arrhythmia, stroke, transient ischemic attack, carotid revascularization, endovascular procedure or surgical intervention for peripheral vascular disease; planned to undergo scheduled PCI, CABG, carotid or peripheral revascularization during the study; systolic blood pressure (BP) >160 mmHg or diastolic BP >100 mmHg at screening visit or randomization visit; history of New York Heart Association (NYHA) Class III or IV heart failure within the past 12 months; known history of hemorrhagic stroke; age <18 years or legal age of majority at the screening visit, whichever is greater; patients not previously instructed on a cholesterol-lowering diet prior to the screening visit; newly diagnosed (within 3 months prior to randomization visit) or poorly controlled (glycated hemoglobin [HbA1c] >8.5% at the screening visit) diabetes; presence of any clinically significant uncontrolled endocrine disease known to influence serum lipids or lipoproteins (note: patients on thyroid replacement therapy can be included if the dosage has been stable for at least 12 weeks prior to screening and between screening and randomization visits, and thyroid-stimulating hormone (TSH) level is within the normal range of the Central Laboratory at the screening visit); history of bariatric surgery within 12 months prior to the screening visit; unstable weight defined by a variation >5 kg within 2 months prior to the screening visit; known history of homozygous or heterozygous familial hypercholesterolemia (FH); known history of loss of function of proprotein convertase subtilisin kexin 9 (PCSK9) (i.e., genetic mutation or sequence variation); use of systemic corticosteroids, unless used as replacement therapy for pituitary/adrenal disease with a stable regimen for at least 6 weeks prior to randomization visit (note: topical, intra-articular, nasal, inhaled, and ophthalmic steroid therapies are not considered as ‘systemic’ and are allowed); use of continuous estrogen or testosterone hormone replacement therapy unless the regimen has been stable in the past 6 weeks prior to the screening visit (week-2) and no plans to change the regimen during the study; history of cancer within the past 5 years, except for adequately treated basal cell skin cancer, squamous cell skin cancer, or in situ cervical cancer; known history of HIV positivity; patients who have taken any investigational drugs other than the alirocumab training placebo kits within 1 month or five half-lives, whichever is longer; patients who have previously participated in any clinical trial of alirocumab or any other anti-PCSK9 therapy; patients who withdraw consent during the screening period (patients who are not willing to continue or fail to return).

Conditions/situations such as: any clinically significant abnormality identified at the time of screening that in the judgment of the investigator or any sub-investigator would preclude safe completion of the study or constrain endpoint assessment such as major systemic diseases or patients with short life expectancy; considered by the investigator or any sub-investigator as inappropriate for this study for any reason, e.g. deemed unable to meet specific protocol requirements, such as scheduled visits; deemed unable to administer or tolerate long-term injections based on the opinion of the patient or investigator; investigator or any sub-investigator, pharmacist, study coordinator, other study staff, or relative thereof directly involved in the conduct of the protocol, etc.; presence of any other conditions (e.g. geographic, social) actual or anticipated which the investigator feels would restrict or limit the patient’s participation for the duration of the study.

Laboratory findings during the screening period (not including randomization assessments): positive test for hepatitis B surface antigen or hepatitis C antibody (confirmed by reflexive testing); positive serum beta-human chorionic gonadotropin (hCG) or urine pregnancy test (including Week 0) in women of childbearing potential; triglycerides >400 mg/dL (>4.52 mmol/L) (one repeat assessment is allowed); eGFR <30 mL/min/1.73 m^2^; alanine aminotransferase (ALT) or aspartate aminotransferase (AST) >3 x upper limit of normal (ULN) (one repeat assessment is allowed); creatine phosphokinase (CPK) >3 x ULN (one repeat assessment is allowed); TSH <lower limit of normal (LLN) or >ULN.

#### Exclusion criteria related to background therapy

All contraindications to the background therapies or warning/precaution of use (when appropriate) as displayed in the respective national product labeling.

#### Exclusion criteria related to alirocumab

Known hypersensitivity to monoclonal antibody therapeutics; pregnant or breast-feeding women; women of childbearing potential with no effective contraceptive method of birth control and/or who are unwilling or unable to be tested for pregnancy. Women of childbearing potential must have a confirmed negative pregnancy test at screening and randomization visits. They must use an effective contraceptive method throughout the study, and agree to repeat urine pregnancy test at designated visits. The applied methods of contraception have to meet the criteria for a highly effective method of birth control according to the note for guidance on non-clinical safety studies for the conduct of human clinical trials and marketing authorization for pharmaceuticals (CPMP/ICH/286/95; <http://www.ema.europa.eu/pdfs/human/ich/028695en.pdf>). Postmenopausal women must be amenorrheic for at least 12 months.

## COMBO II

### Full inclusion criteria

Patients with hypercholesterolemia and established CHD or CHD risk equivalents (see below for definitions) who have poorly controlled LDL-C with a maximally tolerated stable daily dose of statin for at least 4 weeks prior to the screening visit. Signed written informed consent will be obtained from all patients.

Definition of maximally tolerated dose (any of the following are acceptable): rosuvastatin 20 mg or 40 mg daily, atorvastatin 40 mg or 80 mg daily, and simvastatin 80 mg daily (if already on this dose for >1 year - see exclusion criterion). Patients not able to be on any of the above statin doses should be treated with the dose of daily atorvastatin, rosuvastatin, or simvastatin which is considered appropriate for the patient as per the investigator's judgment or concerns. Some examples of acceptable reasons for a patient taking a lower statin dose include: adverse effects on higher doses, advanced age, low BMI, regional practices, local prescribing information, concomitant medications, and comorbid conditions such as impaired glucose tolerance/impaired fasting glucose.

#### Established CHD

Documented history of CHD (includes one or more of the following): acute MI; silent MI; unstable angina; coronary revascularization procedure (e.g. PCI or CABG); clinically significant CHD diagnosed by invasive or non-invasive testing (such as coronary angiography, stress test using treadmill, stress echocardiography, or nuclear imaging).

#### CHD risk equivalents

Definition of CHD risk equivalents includes one or more of the following four criteria:

1. Documented PAD (one of the following criteria [a, b, or c] must be satisfied):
   1. Current intermittent claudication of resumed atherosclerotic origin together with ankle-brachial index equal to or less than 0.90 in either leg at rest OR
   2. History of intermittent claudication together with endovascular procedure or surgical intervention in one or both legs because of atherosclerotic disease OR
   3. History of critical limb ischemia together with thrombolysis, endovascular procedure or surgical intervention in one or both legs because of atherosclerotic disease.
2. Documented previous ischemic stroke with a focal ischemic neurological deficit that persisted more than 24 hours, considered as being of atherothrombotic origin. CT or MRI must have been performed to rule out hemorrhage and non-ischemic neurological disease.
3. Documented CKD as defined by eGFR ≥30‒<60 mL/min/1.73 m^2^ for 3 months or more, including the screening visit.
4. Known history of diabetes mellitus *and* two or more additional risk factors:
   1. History of hypertension (established on antihypertensive medication)
   2. Documented history of ankle-brachial index ≤0.90
   3. Documented history of microalbuminuria or macroalbuminuria or dipstick urinalysis at screening visit (week -3) with >2+ protein.
   4. Documented history of pre–proliferative or proliferative retinopathy or laser treatment for retinopathy.
   5. Known family history of premature CHD (CHD in father or brother before 55 years of age; CHD in mother or sister before 65 years of age).

### Full exclusion criteria

Patients who have met all the above inclusion criteria will be screened for the following exclusion criteria.

#### Exclusion criteria related to study methodology

Patients without established CHD or CHD risk equivalents; LDL-C <70 mg/dL (<1.81 mmol/L) at the screening visit in patients with an history of documented CVD; LDL-C <100 mg/dl (<2.59 mmol/L) at the screening visit in patients without history of documented CVD; change in statin dose or dose regimen from screening to randomization; currently taking a statin that is not simvastatin, atorvastatin, or rosuvastatin; simvastatin, atorvastatin, or rosuvastatin is not taken daily or not taken at a registered dose; daily doses above atorvastatin 80 mg, rosuvastatin 40 mg, or simvastatin 40 mg (except for patients on simvastatin 80 mg for >1 year, who are eligible); use of cholesterol absorption inhibitor (i.e., ezetimibe), omega-3 fatty acid (at doses ≥1000 mg daily), nicotinic acid, bile acid-binding sequestrant, or red yeast rice products within the past 4 weeks prior to screening visit or between screening and randomization visits; use of fibrates in the past 6 weeks prior to screening visit; use of nutraceutical products or over-the-counter therapies that may affect lipids which have not been at a stable dose/amount for at least 4 weeks prior to the screening visit or between screening and randomization visits; patients who have received plasmapheresis treatment within 2 months prior to the screening visit, or have plans to receive it during the study; recent (within 3 months prior to the screening visit or between screening and randomization visits) MI, unstable angina leading to hospitalization, PCI, CABG, uncontrolled cardiac arrhythmia, stroke, transient ischemic attack, carotid revascularization, endovascular procedure or surgical intervention for peripheral vascular disease; planned to undergo scheduled PCI, CABG, carotid or peripheral revascularization during the study; systolic BP >160 mmHg or diastolic BP >100 mmHg at screening visit or randomization visit; history of NYHA Class III or IV heart failure within the past 12 months; known history of hemorrhagic stroke; age <18 years or legal age of majority at the screening visit whichever is greater; patients not previously instructed on a cholesterol-lowering diet prior to the screening visit; newly diagnosed (within 3 calendar months prior to randomization visit) or poorly controlled (HbA1c >9% at the screening visit) diabetes; presence of any clinically significant uncontrolled endocrine disease known to influence serum lipids or lipoproteins (note: patients on thyroid replacement therapy can be included if the dosage has been stable for at least 12 weeks prior to screening and between screening and randomization visits, and TSH level is within the normal range of the Central Laboratory at the screening visit); history of bariatric surgery within 12 months prior to the screening visit; unstable weight defined by a variation >5 kg within 2 months prior to the screening visit; known history of homozygous or heterozygous FH; known history of loss of function of PCSK9 (i.e., genetic mutation or sequence variation); use of systemic corticosteroids, unless used as replacement therapy for pituitary/adrenal disease with a stable regimen for at least 6 weeks prior to randomization visit (note: topical, intra-articular, nasal, inhaled, and ophthalmic steroid therapies are not considered as ‘systemic’ and are allowed); use of continuous estrogen or testosterone hormone replacement therapy unless the regimen has been stable in the past 6 weeks prior to the screening visit and there are no plans to change the regimen during the study; history of cancer within the past 5 years, except for adequately treated basal cell skin cancer, squamous cell skin cancer, or in situ cervical cancer; known history of a positive HIV test; patients who have taken any investigational drugs other than the alirocumab training placebo kits within 1 month or five half-lives, whichever is longer; patients who have been previously treated with at least one dose of alirocumab or any other anti-PCSK9 monoclonal antibody in other clinical trials; patients who withdraw consent during the screening period (patients who are not willing to continue or fail to return).

Conditions/situations such as any clinically significant abnormality identified at the time of screening that in the judgment of the investigator or any sub-investigator would preclude safe completion of the study or constrain endpoint assessment such as major systemic diseases; patients with short life expectancy; considered by the investigator or any sub-investigator as inappropriate for this study for any reason, e.g. deemed unable to meet specific protocol requirements such as scheduled visits; deemed unable to administer or tolerate long-term injections as per the patient or the investigator; investigator or any sub-investigator, pharmacist, study coordinator, other study staff or relative thereof directly involved in the conduct of the protocol, etc.; presence of any other conditions (e.g., geographic, social) actual or anticipated, that the investigator feels would restrict or limit the patient’s participation for the duration of the study.

Laboratory findings during the screening period (not including randomization assessments): positive test for hepatitis B surface antigen or hepatitis C antibody; positive serum beta-hCG or urine pregnancy test (including week 0) in women of childbearing potential; triglycerides >400 mg/dL (>4.52 mmol/L) (one repeat assessment is allowed); eGFR <30 mL/min/1.73 m^2^; ALT or AST >3 x ULN (one repeat assessment is allowed); CPK >3 x ULN (one repeat assessment is allowed); TSH <LLN or >ULN (one repeat assessment is allowed).

#### Exclusion criteria related to the active comparator and mandatory background therapy

All contraindications to ezetimibe or warnings/precautions of use (when appropriate) as displayed in the respective national product labeling; all contraindications to the background statins or warning/precaution of use (when appropriate) as displayed in the respective national product labeling.

#### Exclusion criteria related to alirocumab

Known hypersensitivity to monoclonal antibody or any component of the drug product; pregnant or breast-feeding women; women of childbearing potential not protected by highly effective method(s) of birth control and/or who are unwilling or unable to be tested for pregnancy. Women of childbearing potential must have a confirmed negative pregnancy test at screening and randomization visits. They must use an effective contraceptive method throughout the entire duration of the study treatment, and for 10 weeks after the last intake of study medication (injection or capsule, whichever comes last), and agree to repeat urine pregnancy test at designated visits. (The applied methods of contraception have to meet the criteria for a highly effective method of birth control according to the “Note for guidance on non-clinical safety studies for the conduct of human clinical trials and marketing authorization for pharmaceuticals” [CPMP/ICH/286/95]; http://www.ema.europa.eu/pdfs/human/ich/028695en.pdf). Postmenopausal women must be amenorrheic for at least 12 months).

***Safety assessments during both studies***

Safety will be assessed through adverse event (AE) reporting (including adjudicated cardiovascular events), laboratory analyses, and vital signs measurement. Since the long-term effects of PCSK9 inhibition on top of a statin in humans are unknown, a number of AEs are defined as being of special interest, and will be monitored, including ALT abnormalities, allergic events, hemolytic anemia, pregnancy, overdose with study drug, neurologic events, ophthalmic events, and local injection site reactions. AEs were determined to be of special interest based on review of previous clinical trials, correspondence with regulatory authorities and the Data Monitoring Committee, the use of a monoclonal antibody in this study, and the route of drug administration.

*Safety analyses*

AEs (including adjudicated cardiovascular events), laboratory parameters, and vital signs will be reported descriptively, based on the safety population (all randomized patients who received at least one dose or partial dose of study treatment). The safety analysis will focus on the treatment-emergent AE period defined as the time from the first double-blind dose to the last double-blind dose of the investigational product + 70 days (10 weeks).

Investigators, steering committee and data committee

**Principal Investigators, COMBO I (all USA; National Coordinator in bold)**

**Alexander White (Port Orange, FL)**; Marietta Abalos-Galito (San Jose, CA); Eduardo Almageur (Hialeah, FL); Lawrence Alwine (Dowingtown, PA), Nabil Andrawis (Manassas, VA); Eddie Armas (Hialeah, FL); John Bertsch (Willoughby Hills, OH); William Bestermann (Kingsport, TN); Bradley Block (Oviedo, FL); Cynthia Bowman-Stroud (Paducah, KY); Patricia Buchanan (Eugene, OR); Kristin Bussey-Smith (San Antonio, TX); William Byars (Greer, SC); Christopher Case (Jefferson City, MO); Richard Cherlin (Los Gatos, CA); Bertrand Cole (Newington, NH); Steven Cox (Norman, OK); Charles Dahl (Orem, UT); Matthew Davis (Rochester, NY); Pratik Desai (Cary, NC); Larry Dobkin (Pittsburgh, PA); Timothy Dow (Jonesboro, AR); Daniel Duprez (Minneapolis, MN); Raul Gaona,Sr. (San Antonio, TX); Jeffrey Geohas (Evanston, IL); William Gonte (Southfield, MI); William Graettinger (Sparks, NV); Alan Graff (Ft. Lauderdale, FL); Terry Haas (Vista, CA); James Hampsey (Clearwater, FL); Yehuda Handelsman (Tarzana, CA); David Headley (Port Gibson, MS); Laura Helman (Mishawaka, IN); Angela House (Eagle, ID); Donald Hurley (Charleston, SC); Giovanni Infusino (Chicago, IL); Bernadette Iguh (Houston, TX); Rebecca Jordan (Sacramento, CA); Shahana Karim (Philadelphia, PA); Dean Kereiakes (Cincinnati, OH); Stanley Koch (Morton, IL); James Kopp (Anderson, SC); Larry Kotek (Edina, MN); Michael Ledet (Mobile, AL); Steven Leichter (Columbus, GA); Andrew Lewin (Los Angeles, CA); Tad Lowdermilk (Winston-Salem, NC); Sean Lynd (Cincinnati, OH); Abe Marcadis (Palm Beach, FL); Sashi Makam (New Windsor, NY); Mustafa Mandviwala (Tomball, TX); Gregory McCarroll (San Antonio, TX); Michael McCartney (Methuen, MA); Barry McLean (Homewood, AL); Randall Miller (Eunice, LA); Minesh Patel (Michigan City, IN); Naynesh Patel (Kettering, OH); Marina Raikhel (Lomita, CA); Daniela Renkiewicz (Battle Creek, MI); Jackson Rhudy (Salt Lake City, UT); Brian Riveland (Glendale, AZ); Jeffrey Rosen (Coral Gables, FL); Milroy Samuel (Columbus, OH); Jaime Sandoval (Corpus Christi, TX); Teresa Sligh (Burbank, CA); Cynthia Strout (Mount Pleasant, SC); Phillip Toth (Indianapolis, IN); Pamela Tuck (Montgomery, AL); Ramon Vargas (New Orleans, LA); Gina Vendetti (Boca Raton, FL); Krishnamoorthy Vivekananthan (Houston, TX); Ralph Wade (Bountiful, UT); Franklin Wefald (Smithfield, NC); Debra Weinstein (Boynton Beach, FL); Jonathan Wilson (Winstom Salem, NC); Duane Wombolt (Norfolk, VA).

**Principal Investigators, COMBO II (National Coordinators in bold)**

**Canada: Christian Constance (Montreal, Quebec)**; Naresh Aggarwal (Brampton, Ontario); Anil Gupta (Toronto, Ontario); Guy Tellier (Mirabel, Quebec).

**Denmark: Jorgen Jeppesen (Glostup)**; Jens Brønnum-Schou (Copenhagen); Lars Frost (Silkeborg); Jan Skov Jensen (Hellerup); Kim Klarlund (Køge); Soren Lind-Rasmussen (Hvidovre); Thomas Melchior (Roskilde); Knud Skagen (Herlev); Kristian Korsgaard Thomsen (Esbjerg).

**France: Bertrand Cariou (Nantes);** Eric Renard (Montpellier); Michel Rodier (Nimes); Bruno Verges (Dijon).

**Hungary: Albert Csaszar (Budapest);** Akos Kalina (Budapest); Janos Kis (Budapest); Gyorgy Paragh (Debrecen); Istvan Reiber (Szekesfehervar).

**Israel:** **Dov Gavish (Holon)**; Dror Dicker (Petah Tikva); Ofer Havakuk (Tel Aviv); Osama Hussein (Safed); Hilla Knobler (Rehovot); Michael Lishner (Kfar Saba); Maximo Maislos (Ofakim).

**Russia:** **Alexey Blokhin (Moscow)**; Olga Barbarash (Kemerovo); Svetlana Berns (Moscow); Natalia Burova (St. Petersburg); Galina Chumakova (Barnaul); Marat Ezhov (Moscow); Boris Goloschekin (St. Petersburg); Ivan Gordeev (Moscow); Roman Libis (Orenburg); Olga Orlikova (Saratov); Elena Pavlikova (Moscow); Natalia Polezhaeva (St. Petersburg); Mikhail Sandin (Moscow); Konstantin Sobolev (Moscow); Sergey Yakushin (Ryazan).

**South Africa:** **Dirk Blom (Cape Town);** Lesley Burgess (Cape Town); Eluned Delport (Pretoria); Nyda Fourie (Bloemfontein); Shirley Middlemost (Western Cape); Mohammed Tayob (Middelburg); Hendrik du Toit Theron (Bloemfoentein); Tjaart P Venter (Alberton).

**South Korea:** **Myung-ho Jeong (Gwangjo)**; Kiyuk Chang (Seoul); Ki-Hoon Han (Seoul); Bum-Kee Hong (Seoul); Sang-Ho Jo (Gyeonggido); Moo Hyun Kim (Busan); Sang Hyun Kim (Seoul); Hae-Young Lee (Seoul); Jong-Min Lee (Gyeonggido); Sang-Hak Lee (Seoul); Chang-Wook Nam (Daegu); Jeong Euy Park (Seoul); Seung-Jea Tahk (Gyeonggido); Junghan Yoon (Gangwando).

**Ukraine:** **Ivan Chopey (Uzhhorod);** Oleksandr Karpenko (Kyiv).

**USA:** **Karl Zuzarte (Fall River, MA)**; Mohammed Allaw (Evansville, IN); Vivek Awasty (Marion, OH); Paramvir Bains (Marion, OH); Robert Black (Clearwater, FL); Bradley Block (Oviedo, FL); Eric Bolster (Summerville, SC); David Butuk (Meridian, ID); David Cabrera (Miami, FL); Louis Chaykin (Bradenton, FL); Charles Dahl (Orem, UT); Ronald DeGarmo (Greer, SC); Hugh Durrence (Charleston, SC); Svjetlana Dziko (Lincoln, NE); Mahfouz El Shahawy (Sarasota, FL); John Ervin (Kansas City, MO); Chris Geohas (Phoenix, AZ); Harinder Gogia (Anaheim, CA); Terry Haas (Vista, CA); John Hoekstra (Richmond, VA); Cynthia Huffman (Tampa, FL); Terence Isakov (Lyndhurst, OH); Vicki Kalen (Tuscon, AZ); Norman Lepor (Beverly Hills, CA); Andrew Lewin (Los Angeles, CA); Gerald Lorch (Renton, WA); Irving Loh (Thousand Oaks, CA); Tad Lowdermilk (Winston-Salem, NC); Barry Lubin (Norfolk, VA); Sashi Makam (New Windsor, NY); Amir Malik (Fort Worth, TX); Mustafa Mandviwala (Tomball, TX); Frederick Martin (Bristol, TN); Gilbert Martinez (Chino, CA); Kelli Maw (Brooksville, FL); Barry McLean (Homewood, AL); Roger Miller (Jacksonville, FL); Richard Montgomery (Lexington, NC); Stephen Ong (Oxon Hill, MD); Henry Paez (Miami, FL); Richard Promin (Ocala, FL); Ronald Pruitt (Nashville, TN); Ronald Pucillo (Sugar Land, TX); John Pullman (Butte, MT); Lance Rudolph (Albuquerque, NM); Anthony John Scarsella (Beverly Hills, CA); Richard Schultzaberger (Greenville, NC); Ranjan Shah (Houston, TX); Stephen Thew (Spokane, WA); Gil Vardi (St. Louis, MO); Krishnamoorthy Vivekananthan (Houston, TX); Eric Voth (Topeka, KS); Jeffrey Wayne (Lincoln, CA); Franklin Wefald (Smithfield, NC); Debra Weinstein (Boynton Beach, FL); Matthew D. Wenker (Cincinnati, OH); Alexander White (Port Orange, FL); Daniel Williams (Perrysburg, OH).

**Committees**

**Steering Committee:**

**Chairman:** Henry Ginsberg, MD (Irving Institute for Clinical and Translational Research, Columbia University New York, NY, USA). **Members:** Jennifer G. Robinson, MD, MPH (The University of Iowa, Iowa City, IA, USA); Daniel J. Rader, MD (Institute for Translational Medicine and Therapeutics, University of Pennsylvania School of Medicine Philadelphia, PA, USA); Christopher P. Cannon, MD (Senior Investigator, TIMI Study Group; Professor of Medicine, Harvard Medical School Cardiovascular Division, Brigham and Women's Hospital, Boston, MA, USA); Helen Colhoun, MD, MFPHM (Clinical Centre, University of Dundee, Ninewells Hospital & Medical School, Dundee, UK); John J.P. Kastelein, MD (Academic Medical Center University of Amsterdam, Amsterdam, The Netherlands); Michel Farnier, MD (Le Point Médical, Département d’Endocrinologie et de Lipidologie, Dijon, France).

**Data Monitoring Committee:**

**Chairman:** Anders Olsson, MD (Bromma, Sweden). **Members:** David Waters, MD (Division of Cardiology, San Francisco General Hospital, San Francisco, CA, USA); Dominique Larrey, MD (Hôpital Saint-Eloi Service d'hépato-gastro-entérologie, Montpellier, France); Robert S Rosenson, MD (Director, Cardiometabolic Disorders, Mount Sinai Heart; Professor of Medicine, Mount Sinai School of Medicine, New York, NY, USA); Peter A. Patriarca, MD (Biologics Consulting Group, Inc., Alexandria, VA, USA); Geert Molenberghs, Biostatistician (Center for Statistics (CenStat) Universiteit HasseltAgoralaan 1, Diepenbeek, Belgium).

**Clinical Events Committee (Reviewers), Duke Clinical Research Institute, Durham, NC USA:**

Pierluigi Tricoci, CEC Principal Investigator, Cardiology; Kenneth W Mahaffey, CEC Director, Cardiology; Renato D Lopes, Cardiology; Bimal R Shah, Cardiology; Rajendra H Metha, Cardiology; Matthew T Roe, Cardiology; Zubin Eapen, Cardiology; Luciana Armaganijan, Cardiology; Adriana Bertolami, Cardiology; Sergio Leonardi, Cardiology; Bradley J. Kolls, Neurology; J. Dedrick Jordan, Neurology; Grégory Ducrocq, Cardiology; Etienne Puymirat, Cardiology; Robin Mathews, Cardiology.

List of Institutional Review Boards (IRBs)/Independent Ethics Committees (IECs)

## COMBO I (all USA; most sites covered by National board)

- **National IRB/IEC:**
  - Sterling IRB, Atlanta, GA
- **Local IRBs/IECs (approved some individual sites):**
  - Crescent City IRB, New Orleans, LA
  - Anne Arundel Medical Center (AAMC) IRB, Annapolis, MD
  - Research Subjects' Protection Programs, Minneapolis, MN

## COMBO II (most sites approved by National Boards; some specific sites by Local Boards)

**Canada**

- **National IRB/IEC:**
  - IRB Services, Aurora, Ontario

**Denmark**

- **National IRB/IEC:**
  - De Videnskabsetiske komitéer for Region Hovedstaden Regionsgården, Hillerød

**France**

- **National IRB/IEC:**
  - CPP Ouest IV- Nantes Maison de la Recherche en Santé, Nantes

**Hungary**

- **National IRB/IEC:**
  - Egészségügyi Tudományos Tanács Klinikai Farmakológiai Etikai Bizottsága, Budapest

**Israel**

- **Local IRBs/IECs:**
  - Tel Aviv Sourasky Medical Center, Tel Aviv
  - Rabin Medical Center, Petah-Tikva
  - Meir Medical Center, Kfar Saba
  - Helsinki Committee Kaplan Medical Center, Rehovot
  - Hadassah-Hebrew University Medical Center, Jerusalem
  - Meir Medical Center, Kfar Saba
  - Helsinki Committee Ziv Medical Center, Safed
  - Helsinki Committee Wolfson Medical Center, Holon

**Russia**

- **National IRB/IEC:**
  - Ethics Committee of the Ministry of Healthcare and Social Development of RUSSIAN Federation, Moscow

**South Africa**

- **National IRB/IEC:**
  - Pharma Ethics, Lyttlelton Manor
- **Local IRBs/IECs:**
  - Faculty of Health Services Research Ethics Committee, Cape Town
  - Committee for Pharmaceutical Trials Health Science Faculty University of Stellenbosch, Cape Town
  - University of Pretoria, Faculty of Health Services, Research Ethics Committee, Arcadia, Pretoria
  - Faculty of Health Services, University of the Free State, Bloemfontein

**South Korea**

- **Local IRBs/IECs:**
  - Seoul St. Mary's Hospital IRB, Seoul
  - Yonsei University, Gangnam Severance Hospital IRB, Seoul
  - Hallym University Sacred Heart Hospital IRB, Anyang-si Gyeonggi-do
  - Seoul National University Hospital IRB, Seoul
  - The Catholic University of Korea Uijeongbu St. Mary's Hospital IRB, Uijeongbu City, Gyeonggi-Do
  - Asan Medical Center IRB, Seoul
  - Ajou University Hospital IRB, Suwon
  - Inha University Hospital IRB, Incheon
  - Dong-A University Hospital IRB, Busan
  - Samsung Medical Center IRB, Seoul
  - Yonsei University Health System, Serverance Hospital, Institutional Review Board, Seoul
  - IRB of Chonnam National University Hospital, Gwangju
  - Institutional Review Board of Seoul Metropolitan Government Seoul National University, Seoul
  - Korea University Guro Hospital IRB, Seoul
  - Institutional Review Board of Keimyung University, Dongsan Hospital, Daegu
  - Yonsei University, Wonju College of Medicine, Wonju Christian Hospital Institutional Review Board, Gangwon-do

**Ukraine**

- **Local IRBs/IECs:**
  - Ethics Commission of Administration of Medical Services and Rehabilitation of the State, Kyiv
  - The Ethics Commission of the State Enterprise Clinical Hospital of Uzhhorod, Uzhhorod
  - The Ethics Commission of Outpatient clinic #2 of Shevchenkikvskiy district of Kyiv City, Kyiv
  - The Ethics Commission of Kyiv Municipal Clinical Hospital #1 of the Main Administration of Healthcare in Kyiv, Kyiv
  - The Ethics Commission of Municipal Institution City Hospital #7, Zaporizhzhya

**USA**

- **National IRB/IEC:**
  - Sterling IRB, Atlanta, GA
